# Insights into Palladium Deactivation during Advanced Oxidation Processes

**DOI:** 10.1021/acs.chemmater.2c01951

**Published:** 2022-09-28

**Authors:** Verónica Pinos-Vélez, Oscar Osegueda, Dana Georgiana Crivoi, Jordi Llorca, F. Javier García-García, Mayra G. Álvarez, Francesc Medina, Anton Dafinov

**Affiliations:** † Chemical Engineering Department, Rovira i Virgili University, Av Paisos Catalans 26, 43007Tarragona, Spain; ‡ Departamento de Recursos Hídricos y Ciencias Ambientales, Universidad de Cuenca, Av. 12 de abril,010207Cuenca, Ecuador; § Departamento de Biociencias, Facultad de Ciencias Químicas, Universidad de Cuenca, Av. 12 de Abril, 010207Cuenca, Ecuador; ∥ Centre for Research and Technology Transfer, Universidad Don Bosco, Soyapango, 1874San Salvador, El Salvador; ⊥ Institute of Energy Technologies and Department of Chemical Engineering, Universitat Politècnica de Catalunya, EEBE, 08019Barcelona, Spain; # ICTS-Centro Nacional de Microscopía Electrónica, Universidad Complutense de Madrid, Av. Complutense S/N, 28040Madrid, Spain; ¶ GIR-QUESCAT, Departamento de Química Inorgánica, Facultad de Ciencias Químicas, Universidad de Salamanca, 37008Salamanca, Spain

## Abstract

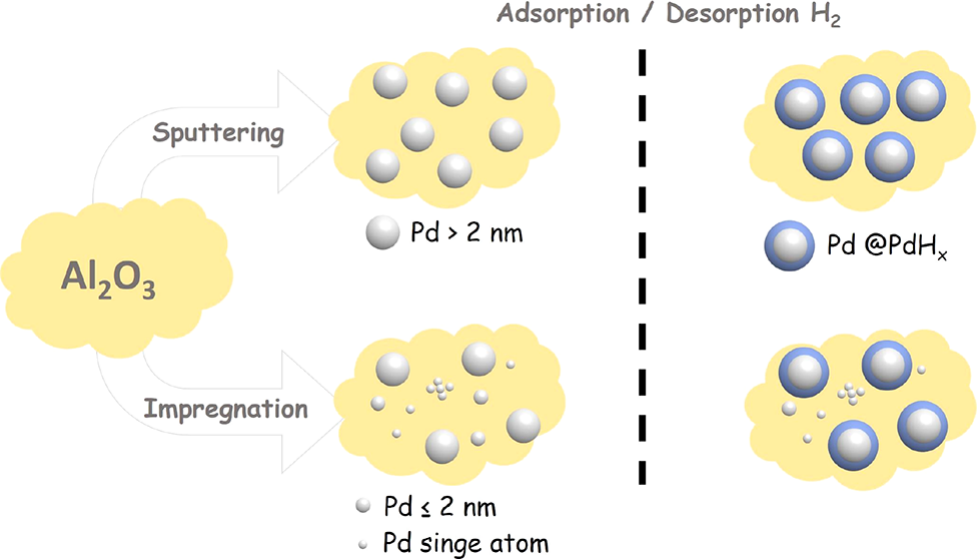

A key step in creating efficient and long-lasting catalysts is understanding their deactivation mechanism(s). On this basis, the behavior of a series of Pd/corundum materials during several hydrogen adsorption/desorption cycles was studied using temperature-programmed desorption coupled with mass spectrometry and aberration-corrected transmission electron microscopy. The materials, prepared by impregnation and by sputtering, presented uniform well-dispersed Pd nanoparticles. In addition, single atoms and small clusters of Pd were only detected in the materials prepared by impregnation. Upon exposure to hydrogen, the Pd nanoparticles smaller than 2 nm and the single atoms did not present any change, while the larger ones presented a core–shell morphology, where the core was Pd and the shell was PdH_*x*_. The results suggest that the long-term activity of the materials prepared by impregnation can be attributed solely to the presence of small clusters and single atoms of Pd.

## 1 Introduction

Known since 1866,^1^ palladium hydrides (PdH_*x*_) have been extensively studied as they serve as an excellent model of solute-driven phase transitions, exhibiting fast kinetics at accessible temperatures and pressures.^2^ The absorption of hydrogen on Pd is governed by three steps: (i) the dissociation of H_2_ into atoms and their chemisorption on the Pd surface, (ii) the diffusion of hydrogen on the Pd surface, and (iii) the diffusion into the bulk metallic structure.^3^ Two distinct phases are formed during the absorption process: an α-phase at a lower hydrogen concentration, where *x* < 0.01, and a β-phase at higher concentrations, *x* ∼0.07,^4−6^ with a boundary line reported at 273 °C and 2.4 MPa at 0.01 < *x* < 0.07.^7^ Interestingly, the phase coexistence occurs only in bulk Pd, while for a single Pd nanocrystal, there is a sudden α-to-β transformation.^8^ While in the bulk PdH_*x*_ the atomic hydrogen is stored in the energetically favored octahedral sites in the face-centered cubic structure, at the nanoscale, there is no change in symmetry but a dilation of the Pd lattice.^9^

The adsorption of hydrogen in the Pd structure induces both elastic and plastic deformations which, in turn, will lead to dislocations in the material; it is considered that these changes are size-dependent.^10^ Griessen *et al.* demonstrated that the hydrogen absorption process on Pd nanoparticles is a coherent process, while the desorption one is mostly incoherent, as in the bulk material.^11^ While the first process does not modify the Pd lattice, during the second one, lattice mismatches are possible. Thus, it comes as no surprise that the interactions between the hydrogen absorption and desorption with Pd will have a strong effect on its catalytic activity.

In our previous work, corundum catalytic membrane reactors (CMRs) containing Pd as the active phase were employed in the *in situ* generation of hydrogen peroxide,^12^ phenol oxidation,^13^ phenol hydrogenation,^14^ chromium(VI) reduction,^15^ and ibuprofen hydrogenation.^14^ In all cases, the Pd-CMRs prepared by impregnation did not lose their catalytic activity after several uses, while the ones prepared by sputtering suffered very fast deactivation. In this study, we investigate the changes that might occur when Pd nanoparticles on corundum powder are exposed to hydrogen. Although both methods produce uniform nanoparticles, the impregnation method leads to single atoms and small clusters which are the main active species. Moreover, we show that the loss in activity is due to the formation of a core–shell palladium hydride structure on the Pd nanoparticles with sizes larger than 2 nm. Although core–shell palladium hydride nanoparticles have been previously reported,^8,16−21^ from the best of our knowledge, this is the first time they are correlated with catalytic deactivation.

## 2 Materials and Methods

### 2.1 Sample Preparations

#### 2.1.1 Method 1—By Impregnation

Palladium was added on the corundum powder using an impregnation method already reported in the literature.^12−15^ In a typical procedure, a known amount of PdCl_2_ (Johnson Matthey, 59.83% Pd) was added, under continuous stirring, to Milli-Q water; HCl (37%, Sigma-Aldrich) was added dropwise to facilitate the dissolution of the metal salt. The impregnation was done in such a way to obtain 2 wt % Pd with respect to the mass of corundum powder. The resulting solid was dried for 5 h at 120 °C, calcined at 450 °C in air, overnight, and reduced under H_2_ (20 standard cubic centimeters per minute—sccm, 3 h at 350 °C). The amount of Pd added was determined from the weight difference between the original and impregnated powder. The material was denoted as Pdi.

#### 2.1.2 Method 2—By Sputtering

A thin layer of corundum powder was placed onto a Petri dish and introduced into the vacuum chamber of a K575X sputter coater (Quorum Technologies). Palladium was pulverized from a Hauner Metallische Werkstoffe palladium target with 95% purity. The background vacuum was set to 10^–5^ Pa, the deposition was carried with pure argon, and the sputtering current was maintained at 30 mA. Three sputtering times were chosen as follows: 30, 90, and 150 s. To determine the amount of Pd deposited on the powder, the procedures were run under the same conditions but using a piece of glass. The thickness of the Pd layer on the glass was calculated from the X-ray reflectometry by the fast Fourier transformation method. All the samples were dried for 2 h at 120 °C; the samples were divided in two: one part was calcined at 350 °C for 6 h and the other one at 600 °C for 6 h; all samples were reduced in hydrogen, 20 sccm for 2 h at 350 °C. The materials were denoted as Pds-*a*-*b*, where *a* represents the sputtering time and *b* the calcination temperature.

### 2.2 Analysis and Characterization

Conventional transmission electron microscopy (TEM) was run on a JEOL model 1011 equipment. Samples were prepared by dispersion in ethanol using sonication and cast onto copper grids coated with carbon mesh. The size of the Pd particles was determined using ITEM Olympus software. High-angle annular dark-field and annular bright-field scanning TEM (HAADF-STEM and ABF-STEM) analyses were run of an aberration-corrected JEM ARM 200 cF equipment. Using these complementary techniques, a better visualization of the potential structural changes that might occur in the samples after hydrogen exposure will be possible, through a better discrimination of the Pd nanoparticles and the Al_2_O_3_ support, since in the HAADF-STEM images, Pd nanoparticles have a higher contrast, while elements with smaller *Z*, such as Al, are easily identifiable in the ABF technique.

X-ray diffraction (XRD) measurements were performed on a Bruker-AXS D8-Discover diffractometer equipped with a parallel incident beam (Göbel mirror), a vertical Θ–Θ goniometer, an *XYZ* motorized stage-mounted Eulerian cradle, diffracted beam Soller slits, and a scintillation counter as a detector. The samples prepared by impregnation were analyzed using an angular step of 0.02° at 47.9 s per step at 25 °C; the angular 2Θ diffraction range was 36.6–44.2° for the samples prepared by impregnation and between 36 and 48° for the ones prepared by sputtering. The X-ray diffractometer was operated at 40 kV and 40 mA to generate Cu Kα radiation (wavelength of 1.54056 Å).

The hydrogen absorption/desorption behavior of Pd was run in an in-house equipment with the following configuration: a tubular furnace, in which a quartz reactor containing the sample is placed vertically; four gas (H_2_, O_2_, Ar, and synthetic air) lines were connected to the upper part of the reactor, and the flow of the gas was controlled using a mass flow controller (Alicat); and the bottom part of the reactor was connected to a mass spectrometer, OmniStar Pfeiffer Vacuum. Typically, two–four cycles of hydrogen saturation were run, followed by hydrogen desorption. Table 1 presents the steps carried out for each cycle.

**Table 1:** Typical Cycle of Hydrogen Adsorption

temperature (°C)	time (min)	flow of H_2_ (sccm)	flow of Ar (sccm)	purpose
60	60	5	45	saturation with H_2_
60	120	0	51.2	purging the lines
60–460	80	0	51.2	temperature-programmed desorption
460	20	0	51.2	elimination of any H_2_ residue in the sample

## 3 Results and Discussion

Our previous studies^12−15^ have shown that the CMRs containing Pd added by sputtering suffered a faster deactivation than those prepared by impregnation. To investigate why this happened, we have prepared Pd/corundum powder materials using the same methods employed for the preparation of the CMRs and analyzed them before and after the hydrogen absorption/desorption cycles. Table 2 presents the percentage of Pd deposited on the powder, along with the mean size of the nanoparticles before the hydrogen absorption/desorption cycles, computed from conventional TEM analysis.

**Table 2:** Weight Percent of Pd and the Mean Size of the Nanoparticles

nr.	material	% Pd with respect to Al_2_O_3_	mean size (nm)^c^
1	Pdi	1.67^a^	12 ± 5
2	Pds-30-350	0.04^b^	5 ± 2
3	Pds-30-600	0.04^b^	7 ± 3
4	Pds-90-350	0.12^b^	6 ± 3
5	Pds-90-600	0.12^b^	14 ± 6
6	Pds-150-350	0.20^b^	8 ± 4
7	Pds-150-600	0.20^b^	13 ± 5

a Determined from the weight difference between the original and modified powder.

b Determined according to method 2 from Section 2.1.

c Determined from conventional TEM analysis.

As expected, increasing the sputtering time will increase the amount of Pd deposited on the corundum powder (Table 2, rows 2–7), slightly affecting the particle mean size (*e.g.*, at 350 °C, Table 2, rows 2, 4, and 6). On the other hand, an increase in the calcination temperature from 350 to 600 °C did favor sintering (Table 2, rows 3, 5, and 7). Although a higher content of Pd was deposited using the impregnation method (Table 2, row 1), the nanoparticles have the same mean size as those obtained from sputtering at exposure times of 90 and 150 s and calcined at 600 °C (Table 2, rows 3, 5, and 7).

**Figure 1:**
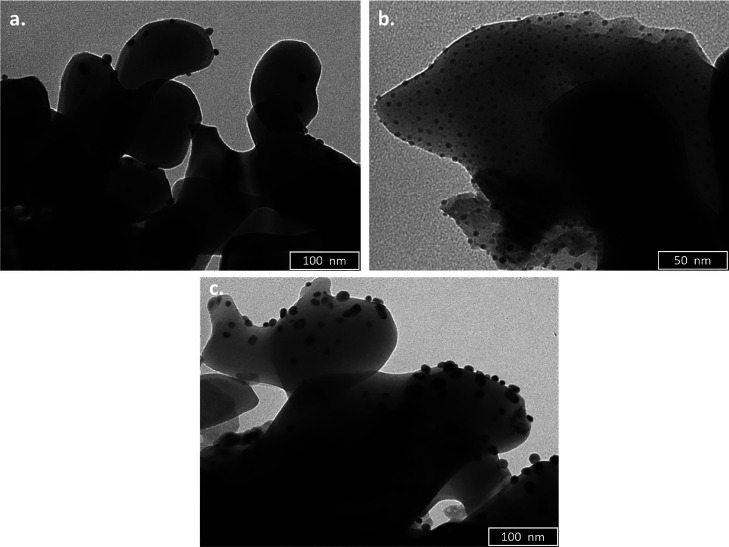
TEM images for (a) Pdi material; (b) Pds-150-350 material, and (c) Pds-150-600 material.

No matter the preparation method (impregnation or sputtering), the Pd nanoparticles have similar morphologies, mainly spherical and well dispersed (Figure 1). As there was no visible difference between the materials prepared using different sputtering times, only the ones prepared for 150 s are presented in this article.

**Figure 2:**
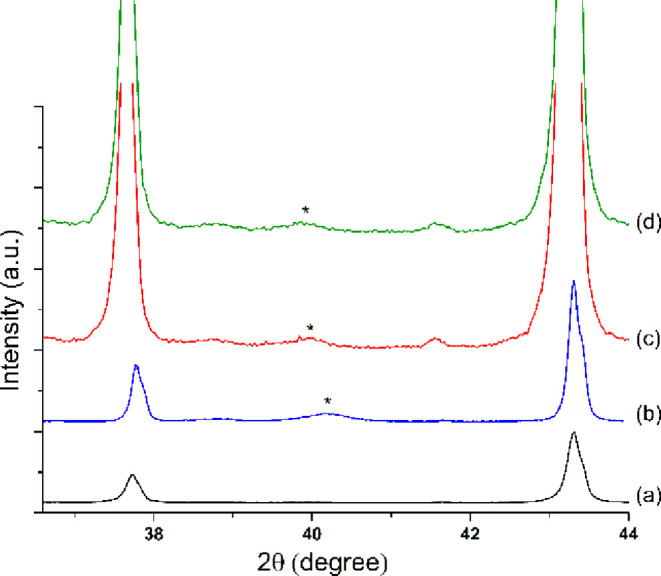
XRD patterns for (a) pure corundum, (b) Pdi, (c) Pds-150-350, and (d) Pds-150-600; *—highlights the peak positions corresponding to the Pd metal (JCPDS #01-088-2335). Diffractograms (c,d) are zoomed in to highlight the peaks corresponding to Pd.

The X-ray diffractograms of all the studied materials present two peaks at 37.7 and 43.3° 2Θ corresponding to the corundum support (Figure 2a). It is well known that XRD is a bulk measurement and the signals are directly connected to the size of the nanoparticles and their concentration in the material.^22,23^ As expected, the diffraction peak at around 40.15°, specific to Pd(111), was clearly visible for the material prepared by impregnation (Figure 2b) and was too weak for the ones prepared by sputtering (Figure 2c,d). In these circumstances, we have only presented the diffractograms of the Pds materials containing the highest content of Pd.

**Figure 3:**
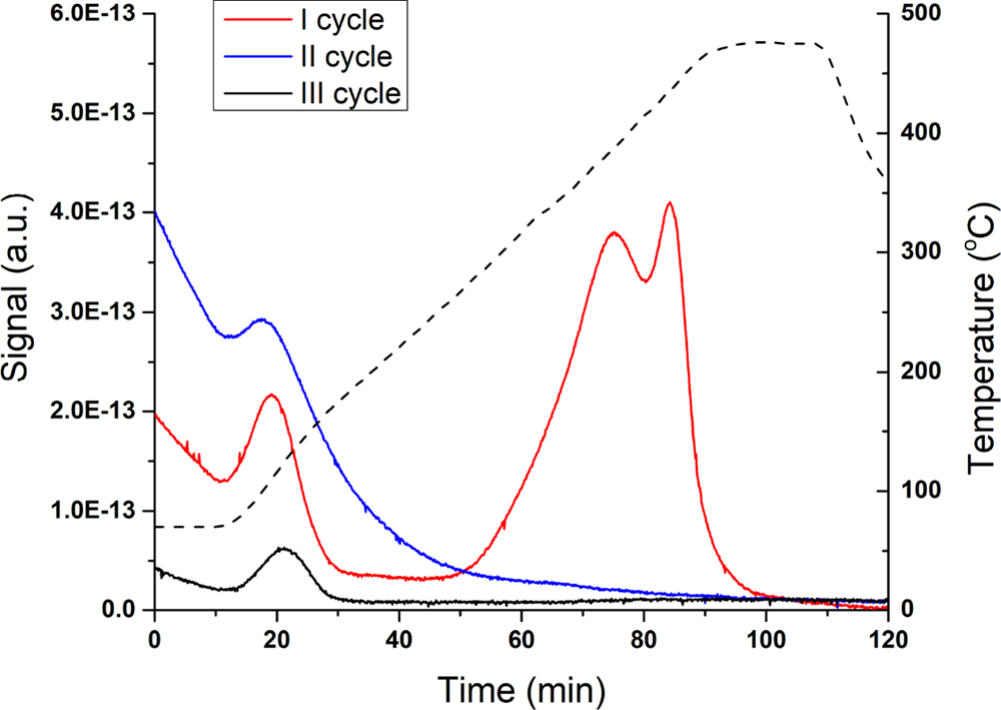
TPD-MS results for the Pdi materials (prepared by impregnation). The dotted line represents the temperature ramp, and its corresponding *Y* axes are in the right.

All materials were subjected to two or three cycles of hydrogen absorption and desorption, as explained in Section 2.2. Previous studies have shown that when Pd is exposed to H_2_, three hydrogen species are formed: a near-surface H, a surface-chemisorbed H, and a bulk-dissolved one. While first species is desorbed at very low temperatures, around −123 °C, the bulk-dissolved one is desorbed at temperatures above 130 °C.^24^ The first cycle run on the Pdi material (Figure 3) presented two main peaks, one at 109 °C attributed to the desorption of the chemisorbed hydrogen and another one, larger, at around 435 °C corresponding to the hydrogen dissolved in the Pd lattice. Surprisingly, when the second and the third cycles were run, no bulk-dissolved hydrogen was detected and only the chemisorbed one was observed. These results indicate that the activity of the CMRs prepared by impregnation is completely attributed to the chemisorbed hydrogen and not the one diffused into the Pd lattice.

**Figure 4:**
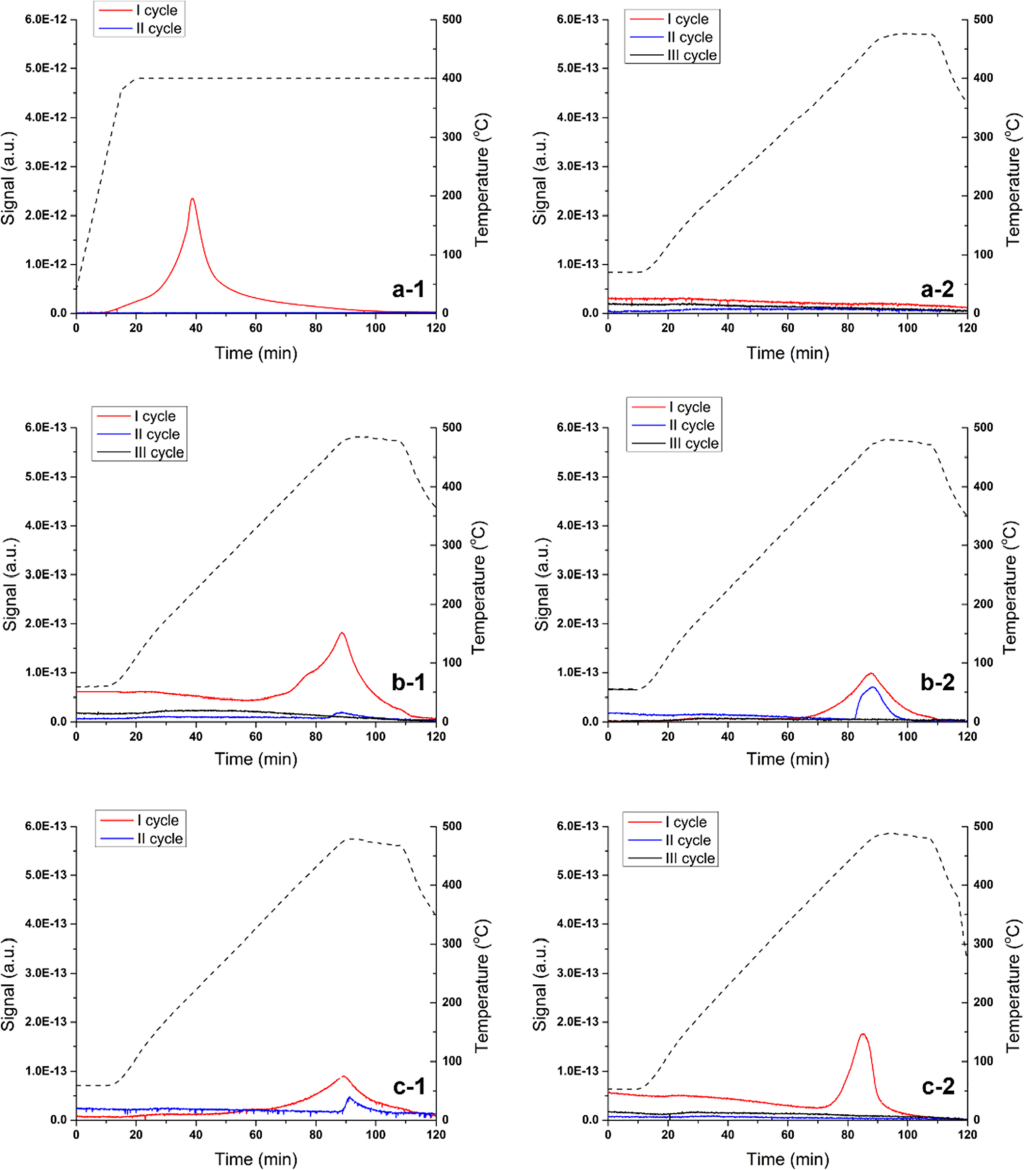
TPD-MS profiles for the materials prepared by sputtering: (a-1) Pds-30-350; (a-2) Pds-30-600; (b-1) Pds-90-350; (b-2) Pds-90-600; (c-1) Pds-150-350; and (c-2) Pds-150-600. The dotted line represents the temperature ramp, and its corresponding *Y* axes are in the right.

The temperature-programmed desorption-mass spectrometry (TPD-MS) profiles of the six materials prepared by sputtering are presented in Figure 4. In the first cycle, all the samples [with the exception of the Pds-30-600 material—Figure 4(a-2)] present the desorption peak corresponding to the bulk-dissolved hydrogen and none of them show the one due to the chemisorbed species. As the bulk-dissolved H cannot exist without the chemisorbed one,^9^ it is safe to consider that the desorption peak corresponding to this H is under the detection limit of the mass detector—which also explains the absence of the peaks for the Pds-30-600 material. After running a second or third cycle of the hydrogen adsorption/desorption process, the peak corresponding to the bulk-dissolved hydrogen is either decreasing or disappearing, demonstrating that the materials are losing their activity. This is in accordance with the catalytic activity observed using the CMRs prepared by the same method.^12−15^ It is important to underline that the possibility of a hydrogen spillover effect is extremely low as less-severe conditions were used in this study compared to the ones where the phenomenon was detected.^25−27^

**Figure 5:**
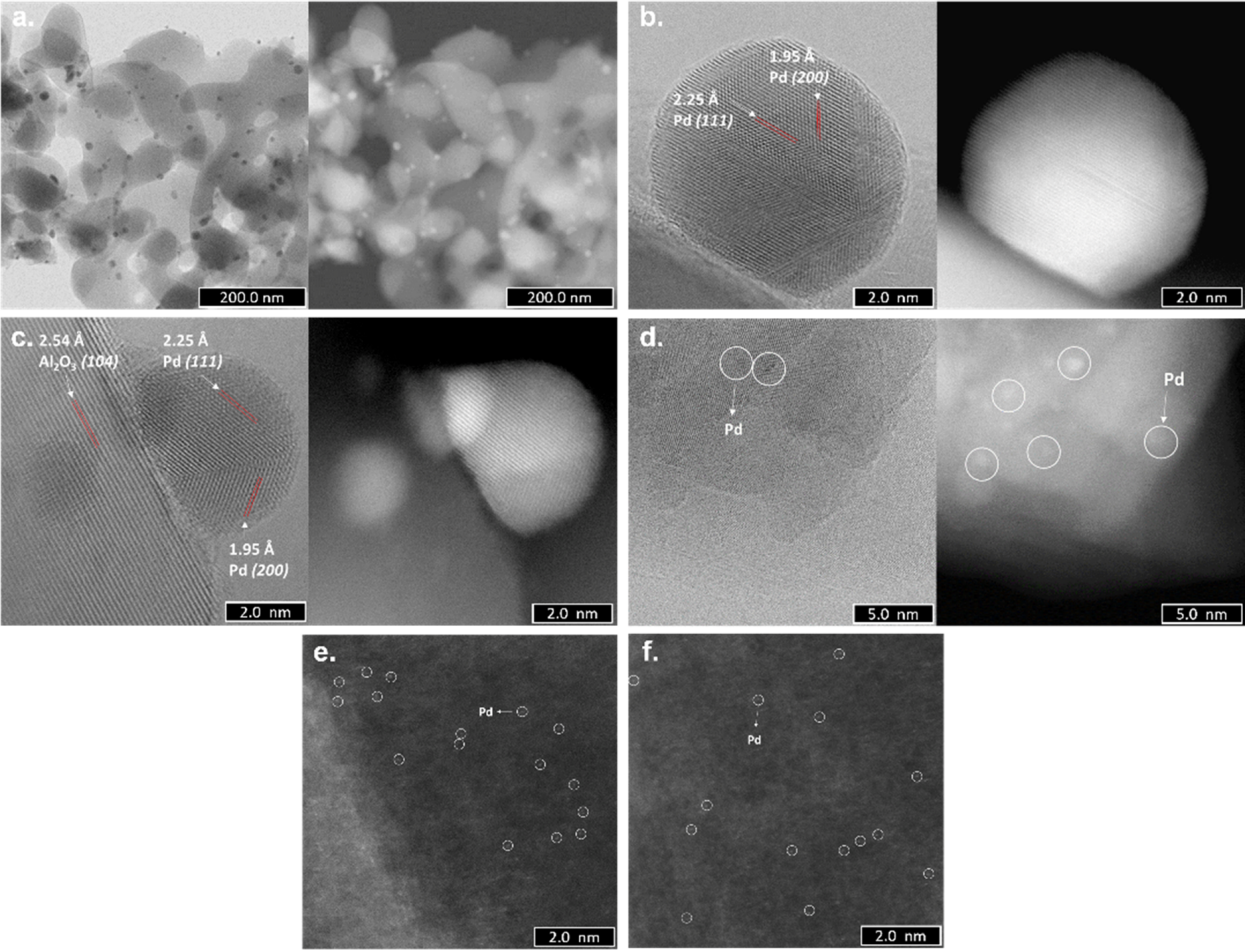
ABF-STEM (left side) and HAADF-STEM (right side) images of the Pdi materials: (a) well-dispersed Pd nanoparticles with sizes between 6 and 10 nm; (b,c) Pd nanoparticles of 1–2 nm; (d) clusters of Pd atoms and HAADF-STEM images; and (e,f) Pd single atoms. For a better visualization, white circles were used.

As the XRD analyses of all materials did not present any visible changes before and after exposure to hydrogen (not shown here), we decided to investigate the materials that showed the most distinctive activity by ABF-STEM and HAADF-STEM: the Pdi and the Pds-30-350. The Pdi material presented very well-dispersed Pd nanoparticles (6–10 nm) (Figure 5a), perfectly crystalline with monodomains with sharp edges. Two lattice fringes with the interplaner distances of 1.95 and 2.25 Å corresponding to the Pd(200) and Pd(111) planes, respectively, were identified in nanoparticles of 1–2 nm in size, along with a lattice fringe of 2.50 Å for the Al_2_O_3_(104) plane^28^ (Figure 5b,c). These indicate the [110] growth direction of the Pd nanoparticles.^29^ In addition, clusters and Pd single atoms were observed, as can be seen in Figure 5d–f.

**Figure 6:**
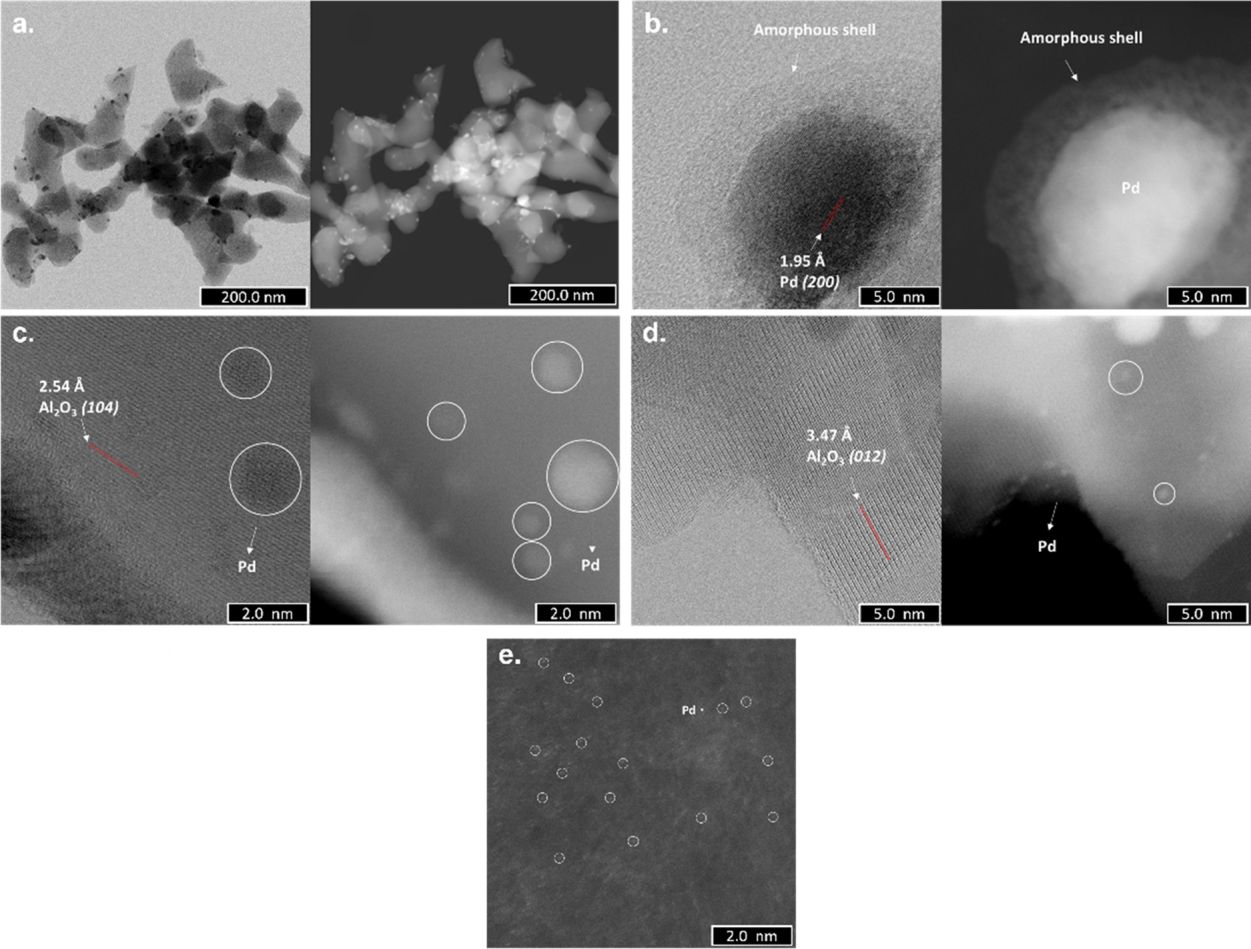
ABF-STEM (left side) and HAADF-STEM (right side) images of the Pdi materials after three cycles of hydrogen adsorption/desorption: (a) well-dispersed Pd nanoparticles with sizes between 6 and 10 nm; (b,c) Pd nanoparticles of 1–2 nm; (d) clusters of Pd atoms and HAADF-STEM images; and – Pd single atoms. For a better visualization, white circles were used. For the Al_2_O_3_ lattice fringes, see ref (30).

After three cycles of hydrogen adsorption and desorption, the large nanoparticles present a core–shell structure, with Pd as the core and an amorphous material as the shell (Figure 6a,b). Intriguingly, this change is not detected for the Pd nanoparticles of 1–2 nm. Moreover, an abundant number of clusters and single atoms of Pd have been detected (Figure 6c–e).

**Figure 7:**
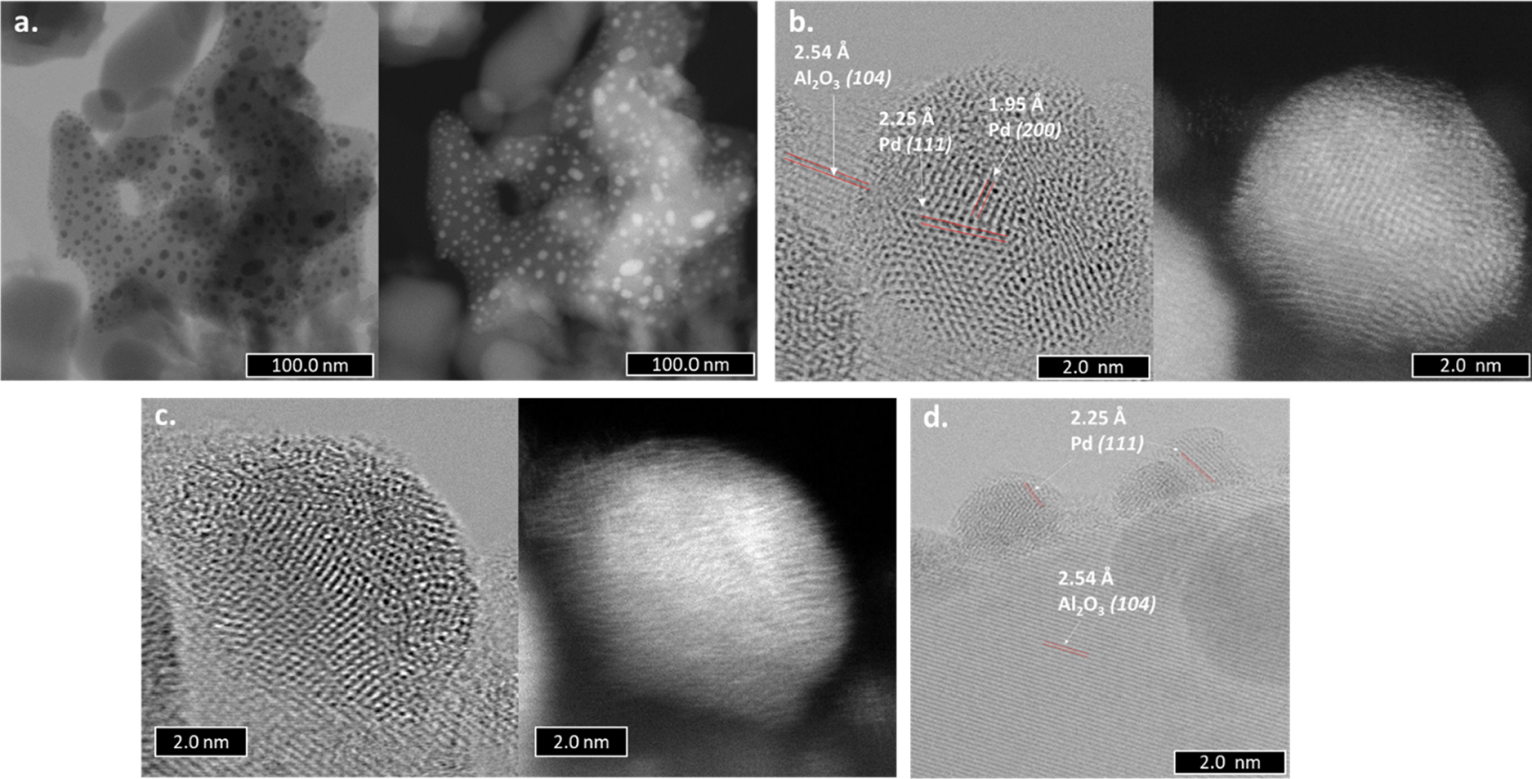
ABF-STEM (left side) and HAADF-STEM (right side) images of the Pds-30-350 materials: (a) poorly ordered Pd nanoparticles and (b–d) (only ABF-STEM) Pd nanoparticles with numerous crystalline domains.

The material prepared by exposure to sputtering for 30 s followed by calcination at 350 °C presents poorly ordered Pd nanoparticles with numerous crystalline domains (Figure 7); these make them highly stressed from a structural point of view. Compared to the Pdi material, in this case, no clusters or single atoms were detected.

**Figure 8:**
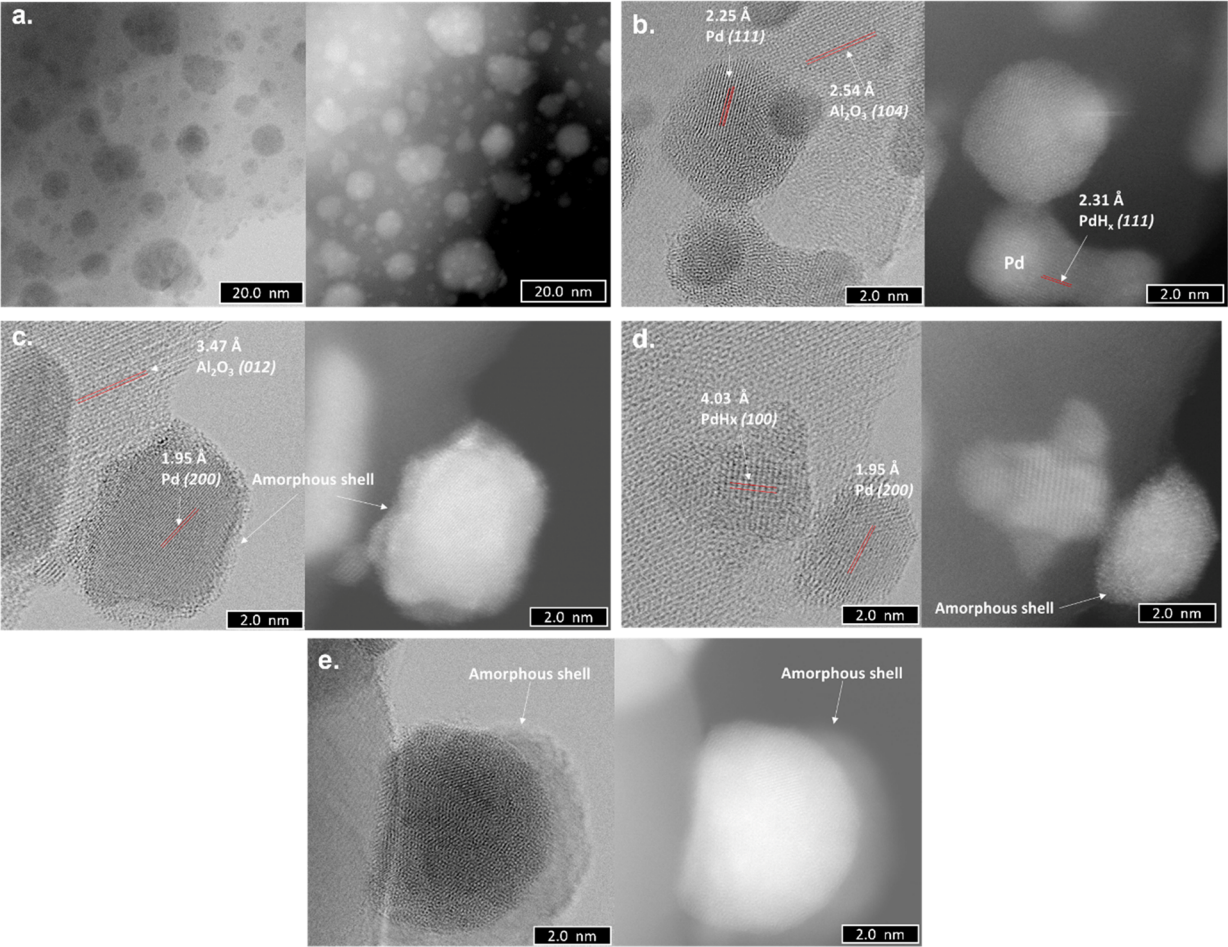
ABF-STEM (left side) and HAADF-STEM (right side) images of the Pds-30-350 materials after exposure to hydrogen: (a,c,e) Pd nanoparticles with a core–shell structure and (b,d) shell corresponding to the palladium hydride.

After one cycle of hydrogen adsorption/desorption, all the Pd nanoparticles found in the Pds-30-350 material present the same core–shell structure as detected in the Pdi ones (Figure 8). In some cases, the shell presented lattice fringes of 2.31 and 4.03 Å corresponding to PdH_*x*_(111) and (100) planes, respectively.^31^ It can be concluded that the disappearance of the peak corresponding to the bulk-dissolved hydrogen after one cycle of H_2_ absorption/desorption process in the TPD-MS profiles for all materials is due to the formation of these core–shell structures.

**Figure 9:**
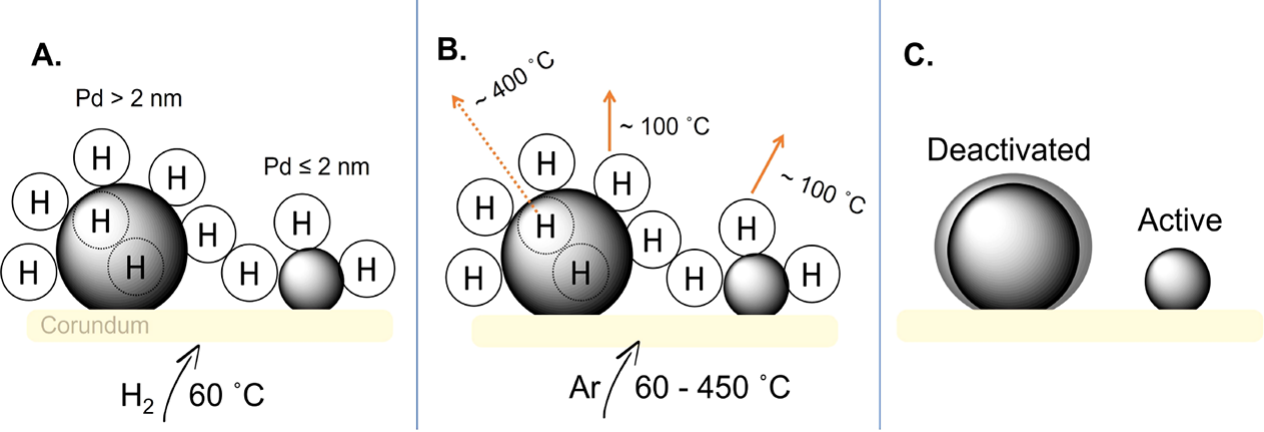
Mechanism of Pd/corundum deactivation.

The hydride formation of Pd is a four-step process, consisting of the following: adsorption of gaseous hydrogen molecules, their dissociation into atoms, penetration of these atoms into the subsurface, and the dissolution in the interstitial sites of the bulk.^32^ It has been already shown that the formation of palladium hydride is directly related with the particle size, where large particles provide more interstitial places for hydride formation, while the smaller ones are prone to chemisorbed hydrogen.^17^ Thus, when Pd/corundum materials prepared by either impregnation or sputtering are exposed for the first time to hydrogen, the particles with sizes larger than 2 nm will be able to absorb and dissolute some of the H atoms (Figure 9A); the penetration of the interstitial sites will cause an expansion of the Pd lattice which, in return, will affect the decomposition of the palladium hydride under the studied conditions. Consequently, during the desorption process, the release of the dissolved hydrogen is accompanied by the formation of a palladium hydride shell which blocks the adsorption of hydrogen in the next cycles (Figure 9B,C). In contrast, the Pd nanoparticles with sizes less than 2 nm are not able to dissolve hydrogen atoms, thus they maintain their activity after several hydrogen adsorption/desorption cycles.

## 4 Conclusions

Based on our previous findings, we have studied the behavior of Pd/corundum materials prepared by impregnation (Pdi) and by sputtering (Pds) under two or three cycles of hydrogen adsorption and desorption using TPD coupled with MS. The results indicate that, after a first cycle, the Pdi material desorbed both the chemisorbed and the interstitial dissolved hydrogen, while the Pds desorbed only the latter one. When subsequent cycles were run, the materials prepared by sputtering presented very low or almost no activity, while for the impregnated materials, only the chemisorbed hydrogen was detected.

Microscopic analysis has showed that the as-prepared Pdi and Pds materials contain well-defined Pd nanoparticles, but the Pdi presented scattered Pd single atoms and clusters of single atoms. Intriguingly, after hydrogen exposure, all Pd nanoparticles with sizes larger than 2 nm exhibited an amorphous palladium hydride shell, while the smaller ones and the single atoms maintained their original structures. The formation of the new core–shell morphology hinders the chemisorption of new hydrogen atoms, thus decreasing the catalytic activity. The findings of the present paper pave the way toward the design of more active Pd catalysts.
